# Oncofetal cell states: cell plasticity drives invasion in premetastatic colorectal tumors

**DOI:** 10.1038/s41392-026-02941-9

**Published:** 2026-08-28

**Authors:** Mahmoud N. A. Amer, Florian R. Greten

**Affiliations:** 1 Institute for Tumor Biology and Experimental Therapy, Georg-Speyer-Haus, Frankfurt/Main, Germany; 2 Department of General, Visceral, Transplant and Thoracic Surgery, Goethe University Frankfurt, Frankfurt, Germany; 3 Frankfurt Cancer Institute, Goethe University Frankfurt, Frankfurt/Main, Germany; 4 German Cancer Consortium (DKTK) and German Cancer Research Center (DKFZ), Heidelberg, Germany

**Keywords:** Cancer microenvironment, Gastrointestinal cancer

In a recent publication in *Nature*, Buissant des Amorie et al. provide evidence that an oncofetal cell state which is associated with advanced disease, chemoresistance and metastatic competence emerges already in early-stage T1 colorectal cancer (CRC). Intriguingly, oncofetal cell states at the invasive front are in close proximity to an exclusive subcluster of cancer-associated fibroblasts (CAFs) thus arguing that cell plasticity towards an oncofetal state is governed by non-cell-autonomous mechanisms.^1^

Oncofetal cells arise when cancer cells hijack the fetal-like regenerative programs normally activated during intestinal injury, acquiring highly plastic cellular states distinct from canonical LGR5⁺ stem cells.^2^ In CRC, oncofetal states exist along a continuum with stem-like states and are shaped by both tumor-intrinsic genetic alterations and signals from the tumor microenvironment.^3,4^ Acquisition of regenerative oncofetal states is increasingly recognized as an important mechanism that enhances metastatic competence, invasion, therapy resistance, and survival under stress. By inducing a slow-cycling phenotype, the oncofetal cell transition confers resistance to antimitotic therapies, enhancing cancer cell survival and increasing the risk of relapse.^5^

Buissant des Amorie et al. now performed spatial transcriptomic profiling of early-stage T1 CRC specimens and identified fetal-like gene expression signatures, including EMP1, MMP7, LAMC2, and ANXA1, at the invasive front, which were associated with a high risk of relapse. The detection of metastasis-associated transcriptional programs in these early-stage tumors was unexpected and led to validation in a larger patient cohort. To this end, the authors analyzed 232 T1 CRC samples and found that the oncofetal cell state was widespread despite the absence of overt metastases, suggesting that oncofetal states arise early during tumor progression, preceding metastatic spread, and suggest that while they may facilitate dissemination, they are not sufficient on their own to drive metastasis. To investigate whether invasive behavior was driven by intrinsic genetic alterations, the authors performed whole-genome sequencing and growth factor dependency assays on organoids derived from oncofetal cells at the invasive front and highly proliferative cells from the tumor core. Both populations harbored identical driver mutations, arguing against the acquisition of distinct genetic alterations or cell-intrinsic features as the basis for invasive properties during early tumor development. Furthermore, pseudo-bulk spatial transcriptomic analyses integrated with single-cell RNA sequencing-derived cell-type signatures identified a relapse-associated tumor microenvironment (TME) and confirmed the attenuation of immune programs linked to metastatic dissemination. Consequently, the authors shifted their focus toward the TME as a key regulator of phenotypic plasticity and immune-evasive behavior.

**Fig. 1:**
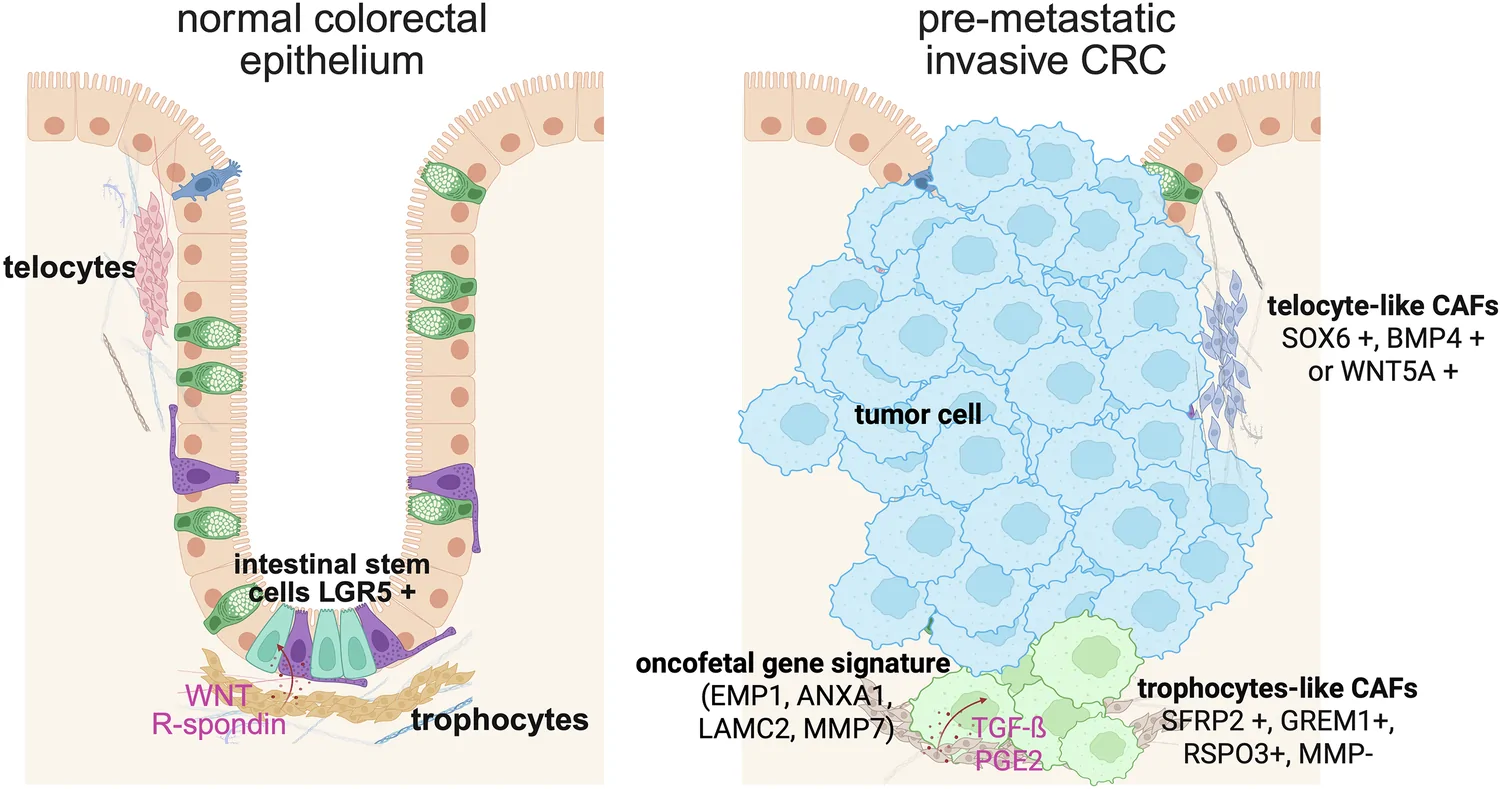
Early induction of oncofetal plasticity during premetastatic colorectal cancer invasion by trophocyte-like cancer-associated fibroblasts. Schematic illustration of the temporal and spatial emergence of oncofetal plasticity during early-stage colorectal cancer (CRC). Stromal remodeling is captured by emergence of FAP+ cancer-associated fibroblasts (CAFs) during progression from normal epithelium to invasive cancer. MMP^+^ trophocyte-like CAFs accumulate at invasive CRC front and secrete TGFβ as well as PGE2 to promote transition of tumor epithelia towards an oncofetal state characterized by expression of EMP1, LAMC2, MMP7 and ANXA1 which is considered to enhance metastatic capacity. Created in BioRender

Single-cell RNA sequencing combined with spatial transcriptomic mapping uncovered stromal heterogeneity associated with cellular plasticity and identified five distinct FAP⁺ cancer-associated fibroblast (CAF) subclusters that were transcriptionally distinct from native intestinal fibroblasts. In the normal intestinal crypt, trophocytes (S3 fibroblasts) are primarily localized at the crypt base, where they interact with LGR5⁺ intestinal stem cells and support niche homeostasis, whereas telocytes (S2 fibroblasts) are predominantly found toward the upper crypt regions. Multiregional spatial transcriptomic analyses revealed a preferential accumulation of trophocyte-like CAFs (SFRP2⁺, GREM1⁺ and RSPO3⁺) at the invasive front, while telocyte-like CAFs (SOX6^+^, BMP4^+^, or WNT5A^+^) were enriched within the tumor core. Among these populations, MMP⁺ trophocyte-like CAFs displayed a particularly strong spatial association with LAMC2⁺ oncofetal cells, suggesting a potential role in supporting invasive tumor cell states (Fig. 1). The authors hypothesized that submucosal trophocytes constitute an early cellular source of trophocyte-like CAFs during CRC progression. Using in vitro co-culture systems, they demonstrated that fibroblasts induce fetal-like reprogramming in both tumor-core- and invasive-front-derived organoids, with the most pronounced effects observed in three-dimensional culture conditions. Moreover, treatment with TGF-β and PGE₂ elicited the strongest upregulation of relapse-associated marker genes, providing compelling evidence that oncofetal plasticity is driven by extrinsic signals through paracrine tumor–stroma crosstalk. Complementary transcriptomic and imaging analyses revealed the emergence of MMP⁺ CAFs immediately following malignant transformation (T1 sm1), with an increasing abundance during disease progression. Notably, MMP⁺ CAFs exhibited close spatial association with LAMC2⁺ oncofetal tumor cells and coincided with localized activation of TGF-β and prostaglandin signaling, implicating these pathways as key regulators of stromal–epithelial interactions that promote invasive cellular states.

Collectively, these findings establish cellular plasticity as a defining feature of early CRC progression, extending beyond tumor cells to encompass stromal compartments of the tumor microenvironment. The study highlights pronounced locoregional differences, with distinct fibroblast populations and oncofetal tumor cell states preferentially enriched at the invasive front, underscoring the importance of the spatial context in shaping tumor plasticity. Rather than representing a uniform ecosystem, the tumor microenvironment appears to consist of specialized niches that differentially influence cellular identity, signaling activity, and invasive potential. The close spatial association between trophocyte-like CAFs and oncofetal tumor cells further suggests that localized stromal–epithelial interactions actively drive phenotypic reprogramming and may establish permissive microenvironments for tumor progression. These observations challenge traditional models that view tumor evolution primarily through the lens of genetic selection and instead support a dynamic framework in which reciprocal interactions between malignant and stromal cell populations continuously reshape cellular states. Such spatially restricted plasticity may enable cancer cells to adapt to changing environmental conditions, thereby fostering heterogeneity, promoting invasion, and ultimately facilitating disease progression. Given that metastatic dissemination and colonization also require adaptation to diverse tissue environments, similar plasticity-driven mechanisms and microenvironmental dependencies may also govern the respective metastatic seeding and niche establishments in different distant organs. However, the precise molecular signals that initiate and sustain these phenotypic transitions remain incompletely defined, while also the contribution of immune cell populations to the induction of oncofetal states remains unclear. Addressing these questions will require functional studies and lineage-tracing approaches to determine the cellular origins, fate trajectories, and dynamic interactions of both tumor and stromal cell populations throughout disease progression and metastatic colonization.
